# Adult neurodevelopmental services in Singapore: A sociodemographic and clinical profile at a tertiary psychiatric hospital

**DOI:** 10.1111/appy.12388

**Published:** 2020-04-14

**Authors:** James Patrick Moon, Ho Teck Tan, Kwok Foong Lam, Jan Mei Lim, Ching Cheng Cheak, Ker‐Chiah Wei, Sreedharan Geetha Sajith, Suet Bin Chai, Giles Ming Yee Tan

**Affiliations:** ^1^ Department of Developmental Psychiatry Institute of Mental Health Singapore; ^2^ Department of Pharmacy Institute of Mental Health Singapore; ^3^ Department of Psychology Institute of Mental Health Singapore; ^4^ Department of Developmental Psychiatry & West Region Institute of Mental Health Singapore; ^5^ Department of Developmental Psychiatry & South Region Institute of Mental Health Singapore; ^6^ Department of Developmental Psychiatry & North Region Institute of Mental Health Singapore; ^7^ Department of Developmental Psychiatry & East Region Institute of Mental Health Singapore

**Keywords:** adult, autism spectrum disorder, intellectual disability, mental retardation, neurodevelopmental disorder

## Abstract

**Introduction:**

The Adult Neurodevelopmental Service in Singapore is the first service of its kind in South‐East Asia for adults with intellectual disability (ID) and/or autism spectrum disorder (ASD). However, few studies have documented and compared the sociodemographic characteristics and clinical needs of this subpopulation group.

**Methods:**

Initial assessments conducted from 1 January 2015 to 31 December 2016 were retrospectively reviewed for this descriptive study.

**Results:**

A total of 272 patients were included in the study (mean age 28.3 ± 11.5; 200 males, 72 females). Adults with ID comprised the largest percentage (52.9%), followed by those with ASD (30.2%), and then those with co‐occurring ASD and ID (16.9%). The ASD subgroup had the highest proportion of individuals with employment, postsecondary school education, functional capabilities, and a psychiatric disorder. In comparison, adults with only ID and adults with co‐occurring ASD and ID shared similar lower levels of education and employment, and had a higher proportion of individuals with epilepsy and aggressive behavior.

**Discussion:**

In this study, adults with ASD had a unique social profile with different clinical needs compared to adults with only ID or to adults with co‐occurring ASD and ID. Adults with only ID and those with co‐occurring ASD shared many of the same social characteristics and high clinical needs. The analysis of these profiles will be useful in developing services that better meet the needs of this complex group.

## 1 INTRODUCTION

Adults with neurodevelopmental disorders (NDDs), such as autism spectrum disorder (ASD) and/or intellectual disability (ID), are a vulnerable and complex group. Individuals with ASD face limitations in social communication and display repetitive or restricted behaviors (American Psychiatric Association, 2013; Wing, 1997). People with ASD also frequently have comorbid ID, which is characterized by significant deficits in intellectual and adaptive functioning (American Psychiatric Association, 2013; Matson & Shoemaker, 2009). Adults with ASD and/or ID also have higher rates of psychiatric and physical comorbidities than the general population (Bregman, 1991; Cawthorpe, 2017; S. A. Cooper et al., 2015; Joshi et al., 2010). Moreover, challenging behavior that arises among children with ASD and/or ID often persists into adulthood (Murphy et al., 2005). One way to address these challenges is through the provision of a neurodevelopmental service for adults composed of multiple disciplines who partner with the patients and their caregivers to provide opportune care (Bouras & Szymanski, 1997).

In 2011, the Adult Neurodevelopmental Service (ANDS) was established at the Institute of Mental Health (IMH) in Singapore. IMH is the only tertiary psychiatric hospital in Singapore. Along with two satellite clinics, IMH serves all regions of Singapore and is readily accessible by public transportation. The ANDS provides both an inpatient and outpatient service for adults with NDD (ASD and/or ID) and is the first service of its kind in South‐East Asia. Every patient is assessed by a multidisciplinary team of a psychiatrist, case manager, psychologist and occupational therapist at the first visit. The services of a medical social worker, nurse and speech therapist are also available if required.

As this is a new service and has not been previously evaluated, there has been little information available on the profile of these adult patients with NDD. For this reason, this study aims to examine and compare the sociodemographic and clinical needs of a cohort of adult patients with ASD and/or ID seen at the ANDS clinic.

## 2 METHODS

### 2.1 Study design and participants

The present study was a descriptive cross‐sectional study conducted at IMH in Singapore. All patients diagnosed with ASD and/or ID who attended the ANDS clinic as a first visit between January 2015 and December 2016 were included in this study. The electronic case records of the initial assessments, containing detailed histories provided by the patient and their caregivers, were accessed. The electronic records were reviewed, and data were collected in relation to the patients' sociodemographic characteristics, NDD characteristics, primary complaints, and psychiatric and medical comorbidities. These variables collected by the clinician at the initial clinic visit were thought to be clinically relevant to guide further interventions and therefore were reported on in this study.

NDD and severity levels of ID along with comorbid psychiatric diagnoses were classified according to DSM‐V categories and were determined by the psychiatrist at the first visit based on clinical assessment from the history given by the patient and/or caregiver. In addition, existing medical or psychiatric conditions may have also been known based on shared electronic records among public hospitals in Singapore and within IMH. Verbal ability, instrumental and basic activities of daily living were assessed by the occupational therapist during the first visit. The Barthel index score was utilized to assist in determining the patients' level of independence. This study was approved by the National Healthcare Group Domain Specific Review Board (DSRB) in Singapore. The DSRB requirements are based on the ethical principles outlined in the Declaration of Helsinki.

### 2.2 Statistical analysis

The data obtained were analyzed using SAS version 9.4. Statistical analysis was performed by applying frequency and percentage tabulation. Chi‐square analysis was used to determine significant differences in categorical variables between the patients' profile characteristics and NDD subgroups. ANOVA and *t* test analyses were used to determine significant differences in mean age among the NDD subgroups. Post hoc analysis was performed using multivariate logistic regression analysis while adjusting for age and gender. The level of statistical significance for all analyses was set at a *P*‐value <.05.

## 3 RESULTS

### 3.1 Neurodevelopmental disorder

A total of 319 patients attended the ANDS new case clinic between January 2015 and December 2016. Of them, 272 patients were accepted by the ANDS department for further follow‐up and received a diagnosis of ASD, ID, or combined ASD with ID, hereafter referred to as “ASD/ID.” Forty‐seven patients did not meet the DSM‐V diagnostic criteria for ID and/or ASD and were not accepted into the service and the study. Patients without a NDD, but were assessed as having a psychiatric condition such as a mood or psychotic disorder were referred to the General Psychiatry department within IMH.

Table 1 shows the diagnostic breakdown of these 272 patients. A total of 52.9% of the patients were assessed as having ID. A total of 30.2% were diagnosed with ASD, and 16.9% had ASD/ID. Among all those diagnosed with ID with or without ASD, 70% were assessed to have a mild level of ID, 25.8% had moderate ID, and 4.2% had severe or profound ID.

**TABLE 1:** Diagnostic breakdown of patients with neurodevelopmental disorders (N = 272)

Diagnosis	N	%
Intellectual disability	144	52.9
Autism spectrum disorder	82	30.2
Combined autism spectrum disorder and intellectual disability	46	16.9
Total	**272**	**100**

### 3.2 Sociodemographic profile

The sociodemographic profile of the patients stratified by the NDD subgroups is detailed in Table 2. Overall, the average age was 28.3 years (SD ± 11.5), with an overall higher proportion of males (73.5%) to females (26.5%). When broken down by the NDD subgroups, the ID subgroup was significantly older than the ASD subgroup (33.5 vs 22.1 years, *t*‐value = 7.8, *P* < .0001) and the ASD/ID subgroup (33.5 vs 23.2 years, *t*‐value = 5.3, *P* < .0001). Compared to the ID subgroup, a significantly higher representation of males was observed among the ASD (85.4% vs 63.2%, *χ* ^2^ = 12.5, df = 1, *P* < .001) and ASD/ID (84.8% vs 63.2%, *χ* ^2^ = 7.5, df = 1, *P* < .01) patient groups. The predominant ethnicity in all the NDD groups was Chinese (77.2%), and most patients were found to be living at home (90.4%).

**TABLE 2:** Sociodemographic profile of neurodevelopmental disorder and its subgroups (N = 272)

Variables	Intellectual disability (N = 144)	Autism spectrum disorder (N = 82)	Combined autism spectrum disorder and intellectual disability (N = 46)	Total (N = 272)	Statistic (*F*‐value or *χ* ^2^)
Age (SD)	33.5 (± 12.6)	22.1 (± 5.4)	23.2 (± 7.4)	28.3(± 11.5)	40.5*
Sex					16.8**
Male	91 (63.2%)	70 (85.4%)	39 (84.8%)	200 (73.5%)	
Female	53 (36.8)	12 (14.6%)	7 (15.2%)	72 (26.5%)	
Ethnic group					Not Significant
Chinese	107 (74.3%)	69 (84.2%)	34 (73.9%)	210 (77.2%)	
Malay	21 (14.6%)	3 (3.7%)	6 (13.0%)	30 (11.0%)	
Indian	11 (7.6%)	5 (6.1%)	4 (8.7%)	20 (7.4%)	
Eurasian or other	5 (3.5%)	5 (6.1%)	2 (4.4%)	12 (4.4%)	
Residence					14.9**
Family home	121 (84.0%)	81 (98.8%)	44 (95.7%)	246 (90.4%)	
Other	23 (16.0%)	1 (1.2%)	2 (4.4%)	26 (9.6%)	
Employment					18.7**
Employment	18 (12.5%)	25 (30.5%)	2 (4.4%)	45 (16.5%)	
Sheltered employment	11 (7.6%)	7 (8.5%)	4 (8.7%)	22 (8.1%)	
Unemployed	115 (79.9%)	50 (61.0%)	40 (87.0%)	205 (75.4%)	
Attendance at a day activity center					17.2**
No	120 (83.3%)	78 (95.1%)	31 (67.4%)	229 (84.2%)	
Yes	24 (16.7%)	4 (4.9%)	15 (32.6%)	43 (5.8%)	
Highest education level					180.9*
Special school/special vocational	97 (67.4%)	15 (18.3%)	41 (89.1%)	153 (56.3%)	
Primary school or less	40 (27.8%)	1 (1.2%)	2 (4.4%)	43 (15.8%)	
Mainstream secondary	3 (2.1%)	10 (12.2%)	2 (4.4%)	15 (5.5%)	
Mainstream postsecondary	4 (2.8%)	56 (68.3%)	1 (2.2%)	61 (22.4%)	

* *P* < .0001.

** *P* < .001.

In the entire sample, the majority were unemployed (75.4%). Only 16.5% of individuals were commercially employed, and 8.1% were employed in a sheltered environment where work skills training was provided. When stratified by the NDD subgroups, while adjusting for age and gender, the ASD patient group had a significantly higher proportion of commercially employed individuals than the ID patient subgroup (30.5% vs 12.5%, *χ* ^2^ = 5.4, df = 1, *P* < .05) and ASD/ID subgroup (30.5% vs 4.4%, *χ* ^2^ = 8.9, df = 1, *P* < .01). The lowest percentages of commercial employment belonged to the ASD/ID subgroup, but did not reach statistical significance when compared to the ID subgroup (*P* = .08).

Among all the NDD patients, only 5.8% attended day activity centers in the community. The lowest attendance belonged to the ASD subgroup, and was significantly lower when compared to the ID subgroup (4.9% vs 16.7%, *χ* ^2^ = 8.4, df = 1, *P* < .01) and the ASD/ID subgroup (4.9% vs 32.6%, *χ* ^2^ = 14.3, df = 1, *P* < .001). The proportion of those attending a day activity center did not differ significantly between the ID and ASD/ID subgroups (*P* = .18).

With regard to the highest education level achieved, the majority (56.3%) had attended special schools with curriculum designed for those with NDD in Singapore. When broken down by the NDD subgroups while adjusting for age and gender, the ASD patient group was found to have a significantly higher proportion of individuals who had postsecondary school education vs those with ID (68.3% vs 2.8%, *χ* ^2^ = 44.1, df = 1, *P* < .0001) and to those with ASD/ID (68.3% vs 2.2%, *χ* ^2^ = 19.4, df = 1, *P* < .0001). The proportion of individuals with postsecondary school education did not differ significantly between the ID and ASD/ID subgroups (*P* = .52). The ASD/ID subgroup had the highest proportion of individuals attending special schools (89.1%) which did not differ significantly with those with ID (67.4%, *P* = .4), but were both found to be significantly higher than those with ASD (18.3%, *P* < .001).

### 3.3 Referral source and primary complaint

Table 3 shows the breakdown of the referral source and the primary presenting complaint at the first visit to the ANDS clinic. The majority of patients (70.2%) were referred internally from other departments within IMH such as from the department of child psychiatry or general psychiatry. At IMH, patients with a NDD who have turned 19 years of age in the department of child psychiatry will be referred to ANDS for further follow‐up.

**TABLE 3:** Referral source and primary complaint and its subgroups (N = 272)

Variables	Intellectual disability (N = 144)	Autism spectrum disorder (N = 82)	Combined autism spectrum disorder and intellectual disability (N = 46)	Total (N = 272)	Statistic (*χ* ^2^)
Referral source					Not significant
Internal referrals	99 (68.8%)	59 (72.0%)	33 (71.7%)	191 (70.2%)	
External referrals from hospitals, clinics, or general practitioners	17 (11.8%)	3 (3.7%)	2 (4.4%)	22 (8.1%)	
Self‐referral	28 (19.4%)	20 (24.4%)	11 (23.9%)	59 (21.7%)	
Primary complaint					71.9*
Aggressive behavior towards others or objects	78 (54.2%)	16 (19.5%)	23 (50.0%)	117 (43.0%)	
Self‐injurious behavior	13 (9.0%)	0 (0.0%)	7 (15.2%)	20 (7.4%)	
Mood or psychotic symptoms	14 (9.7%)	13 (15.9%)	2 (4.4%)	29 (10.7%)	
Transition from child services or seeking diagnosis	5 (3.5%)	32 (39.0%)	8 (17.4%)	45 (16.5%)	
Resources to help improve social and interpersonal skills or daily living skills.	25 (17.4%)	15 (18.3%)	4 (8.7%)	44 (16.2%)	
Other	9 (6.2%)	6 (7.3%)	2 (4.3%)	17 (6.2%)	

* *P* < .0001.

Overall, aggressive behavior was the most common reported primary complaint (43.0%). Compared to their ASD counterparts, aggressive behavior was more common among patients with ID (54.2% vs 19.5%, *χ* ^2^ = 19.4, df = 1, *P* < .0001) and among those with ASD/ID (50.0% vs 19.5%, *χ* ^2^ = 12.3, df = 1, *P* < .001). The proportion of individuals with aggressive behavior was not significantly different between the ID and ASD/ID subgroups (*P* = .6). The most common presentation for the ASD subgroup was for seeking a diagnosis or transition from child services within IMH (39.0%). Among all NDD subgroups overall, the second and third most common primary complaint was for seeking a diagnosis or transition from child services within IMH (16.5%) and for resources to help improve social and interpersonal skills or daily living skills (16.2%), respectively. Examples of other presenting complaints included side effects, transfer of care from private care or other departments, nursing home placements, assessments for fitness to work, applications for deputyship, or poor sleep.

### 3.4 Clinical profile: functional status and psychiatric and physical comorbidity

Table 4 shows the functional capabilities across the NDD subgroups with regard to basic and instrumental activities of daily living and verbal abilities. The ASD subgroup was the most fully independent in their basic activities of daily living, with 95.1% being independent compared to only 56.2% in the ID subgroup (*χ* ^2^ = 17.6, df = 1, *P* < .0001) and 45.7% in the ASD/ID subgroup (*χ* ^2^ = 28.2, df = 1, *P* < .0001). The ASD subgroup also had a significantly higher proportion of individuals who were independent in their instrumental activities of daily living than the ID subgroup (76.8% vs 21.5%, *χ* ^2^ = 35.3, df = 1, *P* < .0001) and ASD/ID subgroup (76.8% vs 15.2%, *χ* ^2^ = 35.5, df = 1, *P* < .0001). The ASD subgroup also displayed significantly higher verbal abilities, with 94.4% being able to speak in full sentences, compared to only 63.2% in the ID group (*χ* ^2^ = 15.5, df = 1, *P* < .0001) and 52.2% in the ASD/ID group (*χ* ^2^ = 23.2, df = 1, *P* < .0001).

**TABLE 4:** Clinical profile of neurodevelopmental disorder and its subgroups (N = 272)

Variables	Intellectual disability (N = 144)	Autism spectrum disorder (N = 82)	Combined autism spectrum disorder and intellectual disability (N = 46)	Total (N = 272)	Statistic (*χ* ^2^)
Basic activities of daily living					45.7*
Fully independent	81 (56.2%)	78 (95.1%)	21 (45.7%)	180 (66.2%)	
Partially or totally dependent	63 (43.8%)	4 (4.9%)	25 (54.3%)	92 (33.8%)	
Instrumental activities of daily living					91.8*
Fully independent	31 (21.5%)	63 (76.8%)	7 (15.2%)	101 (37.1%)	
Partially dependent	75 (52.1%)	19 (23.2%)	34 (73.9%)	128 (47.1%)	
Totally dependent	38 (26.4%)	0 (0%)	5 (10.9%)	43 (15.8%)	
Verbal communication					42.5*
Nonverbal	20 (13.9%)	2 (2.4%)	13 (28.3%)	35 (12.9%)	
Partial	33 (22.9%)	1 (1.2%)	9 (19.5%)	43 (15.8%)	
Full	91 (63.2%)	79 (96.3%)	24 (52.2%)	194 (71.3%)	
Comorbid psychiatric disorder					6.7***
Yes	38 (26.4%)	34 (41.5%)	11 (23.9%)	83 (30.5%)	
No	106 (73.6%)	48 (58.5%)	35 (76.1%)	189 (69.5%)	
Schizophrenia and other psychotic disorders					Not significant
Yes	21 (14.6)	6 (7.3%)	3 (6.5%)	30 (11.0%)	
No	123 (85.4%)	76 (92.7%)	43 (93.5%)	242 (89.0%)	
Anxiety disorders					15.1**
Yes	4 (2.8%)	14 (17.1%)	3 (6.5%)	21 (7.7%)	
No	140 (97.2%)	68 (82.9%)	43 (93.5%)	251 (92.3%)	
Attention deficit hyperactive disorder (ADHD)					10.9***
Yes	4 (2.8%)	12 (14.6%)	4 (8.7%)	20 (7.4%)	
No	140 (97.2%)	70 (85.4%)	42 (91.3%)	252 (92.6%)	
Depressive disorders					Not significant
Yes	9 (6.3%)	9 (11.0%)	1 (2.2%)	19 (7.0%)	
No	135 (93.7%)	73 (89.0%)	45 (97.8%)	253 (93.0%)	
Comorbid physical condition					17.3**
Yes	77 (53.5%)	21 (25.6%)	17 (37.0%)	115 (42.3%)	
No	67 (46.5%)	61 (74.4%)	29 (63.0%)	157 (57.7%)	
Epilepsy					13.9**
Yes	24 (16.7%)	1 (1.2%)	9 (19.6%)	34 (12.5%)	
No	120 (83.3%)	81 (98.8%)	37 (80.4%)	238 (87.5%)	
Skin conditions					Not significant
Yes	15 (10.4%)	9 (11.0%)	3 (6.5%)	27 (9.9%)	
No	129 (89.6%)	73 (89.0%)	43 (93.5%)	245 (90.1%)	

* *P* < .0001.

** *P* < .001.

*** *P* < 0.05.

Table 4 also displays the proportion of the NDD patients with a comorbid psychiatric disorder and physical condition. Overall, the ASD subgroup had a significantly higher proportion of patients with a comorbid psychiatric disorder than the ID group (41.5% vs 26.4%, *χ* ^2^ = 3.8, df = 1, *P* < .05) and the ASD/ID group (41.5% vs 23.9%, *χ* ^2^ = 3.9, df = 1, *P* < .05). The proportion of individuals with a comorbid psychiatric disorder was not significantly different between the ID and ASD/ID subgroups (*P* = .7). The most common psychiatric conditions affecting the ASD group were anxiety disorders (17.1%). When compared to adults with ID, the prevalence of anxiety disorders in those with ASD was found to be significantly higher (17.1% vs 2.8%, *χ* ^2^ = 4.2, df = 1, *P* < .05), and this difference in prevalence almost reached statistical significance when compared to the ASD/ID group (17.1% vs 6.5%, *P* = .1). Schizophrenia and psychotic disorders were the most common comorbid psychiatric conditions among those with ID (14.6%). Among the ASD/ID group, schizophrenia (6.5%) and anxiety disorders (6.5%) were the two most common psychiatric comorbidities.

The ID subgroup overall had the highest proportion of adults with a comorbid physical condition (53.5%), and when adjusted for age and gender this proportion was significantly higher than the ASD subgroup which had the lowest percentage (53.5% vs 25.6%, *χ* ^2^ = 7.6, df = 1, *P* < .01). The proportion of individuals with a comorbid physical condition was not significantly different between the ID and ASD/ID subgroups (53.5% vs 37.0%, *P* = .4). The most common physical condition affecting the ID subgroup (16.7%) and the ASD/ID subgroup (19.6%) was epilepsy. Compared to their ASD counterparts, the prevalence of epilepsy was found to be significantly higher in the ID subgroup (16.7% vs 1.2%, *χ* ^2^ = 7.0, df = 1, *P* < .01) and the ASD/ID group (19.6% vs 1.2%, *χ* ^2^ = 7.7, df = 1, *P* < .01). The proportion of individuals with epilepsy between the ID and ASD/ID was not significant (*P* = .7). The most common physical conditions affecting the ASD group were skin‐related conditions (11.0%).

## 4 DISCUSSION

This study aimed to determine the sociodemographic and clinical profile of adult patients accessing the ANDS clinic at IMH in Singapore. In the entire sample of those with NDD, ID comprised the largest percentage (52.9%), followed by ASD (30.2%), and then ASD/ID (16.9%). The rates of individuals with co‐occurring ASD/ID in our study were consistent with other studies, which estimated that 28% of ID cases have co‐occurring ASD (Bryson, Bradley, Thompson, & Wainwright, 2008). The male predominance among the patients with ASD and ASD/ID was consistent with figures from previous literature in which ASD typically affects males more than females at a 4:1 ratio (Baron‐Cohen et al., 2011). Moreover, individuals with ASD or combined ASD/ID were found to be younger than those with ID. Due to this difference in age and gender among the NDD subgroups, multivariate logistic regression analysis was employed in this study to control for them. With regards to ethnicity, the majority of the patients in the study were of Chinese ethnicity (77.2%), followed by Malay (11.0%) and Indian (7.4%), which largely reflects the demographic profile of Singapore, where the majority is Chinese (74.3%), followed by Malays (13.4%), and then Indians (9.0%) (Singapore Department of Statistics, 2018).

What is evident in this study is the emergence of two distinct social profiles with different clinical needs—the ID and ASD/ID groups shared similar social characteristics and clinical needs vs their distinct ASD counterparts. Compared to the ASD subgroup, the ID and ASD/ID subgroups had larger percentages of individuals attending special schools and day activity centers, and were the least likely to be employed. This finding suggests that despite their attendance at special schools, individuals with ID or combined ASD/ID have different experiences and access to employment services than individuals with ASD alone. Previous literature has shown that lower cognitive abilities among individuals with ASD is a significant predictor for poorer work outcomes (Holwerda, van der Klink, Groothoff, & Brouwer, 2012). These findings suggest the need for adequate support from systems and professionals for integrated employment for those with ID.

Furthermore, the ID subgroup had the highest proportion of individuals with a comorbid physical condition. This finding is consistent with the literature that has shown that adults with ID face high rates of comorbid medical conditions, highlighting the need for focused health care services for this subpopulation (S. A. Cooper et al., 2015).

Moreover, the presence of ID, whether co‐occurring with ASD or not, was associated with lower verbal abilities, higher rates of epilepsy, and a higher likelihood of presenting with aggressive behavior. This is in line with previous studies that have shown that as IQ decreases, challenging behaviors increase, making ID a major risk factor (S.‐A. Cooper et al., 2009; Mcclintock, Hall, & Oliver, 2003). Prior studies have found that communication deficits are common among those with ID and are associated with challenging behavior (Belva, Matson, Sipes, & Bamburg, 2012; Deb, Thomas, & Bright, 2002; Mcclintock et al., 2003). Previous studies have also shown that epilepsy is highly prevalent among those with ID and may be associated with severe behavioral problems (Bowley & Kerr, 2000; Deb et al., 2002). It is recommended that future research explore how epilepsy may play a mediating role in the manifestation of challenging behavior among those with ID.

Conversely, individuals with ASD without ID emerged with the most notable differences when compared to other NDD subgroups. Those with ASD had the highest rates of commercial employment, functional capabilities, and the highest levels of education. These individuals were the least likely to present with aggressive behavior and were the least likely to have a comorbid physical condition and epilepsy. These figures are not surprising given that severity levels can be heterogeneous among those with ASD, who range from low‐ to high‐functioning individuals (Masi, DeMayo, Glozier, & Guastella, 2017). People with ASD who have higher levels of education and greater adaptive skills will likely have greater capacities in accessing health care and employment resources and will be the least likely to present with challenging behavior. The ASD subgroup, however, had a significantly higher proportion of individuals with a comorbid psychiatric disorder, specifically anxiety disorders. This finding is consistent with previous literature whereby caregivers have reported comorbid psychiatric conditions in up to 34.1% of adults with ASD, for which anxiety diagnoses were the most common (Buck et al., 2014). Overall, higher rates of comorbid psychiatric conditions in the ASD subgroup may also be in part attributed to the difficulty in diagnosing psychopathology among those with ID due to its atypical presentation and relative difficulty in eliciting symptoms among those with verbal deficits (Verhoeven & Tuinier, 1997).

### 4.1 Limitations

This study was a cross‐sectional study; therefore, causality cannot be established. Moreover, due to ethical considerations, there were limitations in accessing electronic health records with other health care providers; therefore, the true burden of medical diseases in this study population may have been underreported. Furthermore, being a study at a tertiary psychiatric hospital in Singapore with a high volume of internal referrals, it has the limitation of not being representative of all adults with NDD in Singapore. For instance, the ASD and ASD/ID subgroups were found to be younger than the ID subgroup. This overrepresentation of younger adults in the ASD and ASD/ID subgroups likely reflects a sampling bias arising from the large number of referrals internally from IMH's child psychiatry department which offers a service for children up to 18 years affected by ASD with and without ID, but not those with only ID. IMH also receives referrals from primary and secondary care services from across the country and is readily accessible by public transportation. The patient population attending the ANDS would be likely geographically representative of the country.

Although the patients' education and employment were used as proxies, measurements of socioeconomic status (SES) could have been improved by ascertaining the patients' families' education, income, composition and occupation. In this study, data were retrospectively retrieved from the patients' first visit to the ANDS clinic. The first visit however primarily focused on the clinical assessment of the patient and therefore, key SES indicators of the patients' families or caregivers were not collected.

In the literature, the relationship between SES and the diagnosis of NDD has been shown to be significantly associated. For instance, evidence suggests that families raising a child with ID or combined ASD/ID often do so in a context of social and economic disadvantage (Delobel‐Ayoub et al., 2015; Fujiura, 1998; Fujiura & Yamaki, 1997). For ASD, studies yielded conflicting results. In the United States, families with high SES was positively associated with ASD (Bhasin & Schendel, 2007; Bilder, Pinborough‐Zimmerman, Miller, & McMahon, 2009; Croen, Grether, & Selvin, 2002; Windham et al., 2011). However, it has been suggested these associations primarily reflect a bias in case ascertainment due to SES inequalities in accessing health care services (Rai et al., 2012). On the contrary, European studies found associations between low SES with ASD (Emerson, 2012; Larsson et al., 2005; Rai et al., 2012).

Given that families accessing the ANDS clinic would be eligible to receive governmental financial assistance if needed, financial barriers accessing ANDS would likely be minimized. Therefore, consistent with studies in Europe, patients and their families would also likely be of lower SES than that of the general population who utilize ANDS in Singapore. However, future studies in non‐Western countries like Singapore would help elucidate further the relationship between SES, ASD, and ID in an Asian context.

Finally, this study combined high‐ and low‐functioning ASD patients for analysis. High‐ and low‐functioning ASD patients, however, likely represent two distinct subpopulations that have key differences in prevalence, associations with challenging behaviors, and comorbid medical and psychiatric conditions. Further studies are warranted to explore these associations with consideration of functional abilities among adults with ASD.

## 5 CONCLUSION

The current study suggests that adults with only ASD who utilize the ANDS in Singapore have a distinctive social profile with different clinical needs compared to adults with ID or to adults with combined ASD and ID. In particular, psychiatric care was a clear need for the ASD subgroup who presented with a higher proportion of individuals suffering from a comorbid psychiatric condition. On the other hand, adults with only ID and adults with combined ASD and ID shared many of the same social characteristics and high clinical needs. They presented with similar lower levels of education and employment, and higher proportion of individuals with epilepsy and aggressive behavior. To adequately address the needs of this subpopulation, will therefore require a multidisciplinary service in assessing and managing their mental health, physical and social needs. Overall, the similarities and differences highlighted in this study among adults with NDD should be taken into account in order to improve services to better meet the needs of this complex group.

## AUTHOR CONTRIBUTIONS

J. P. M., H. T. T.: Acquisition, analysis, and interpretation of data; drafting the work and revising it critically for important intellectual content; final approval; agreement to be accountable for all aspects of the work. K. F. L., J. M. L., C. C. C.: Acquisition, analysis of data; drafting the work and revising it critically for important intellectual content; final approval; agreement to be accountable for all aspects of the work. K.‐C. W., S. G. S., S. B. C.: Interpretation of data; drafting the work and revising it critically for important intellectual content; final approval; agreement to be accountable for all aspects of the work. G. M. Y. T.: Conception and design of the work; interpretation of data; drafting the work and revising it critically for important intellectual content; final approval; agreement to be accountable for all aspects of the work.

## Data Availability

The data that support the findings of this study are available on request from the corresponding author. The data are not publicly available due to privacy or ethical restrictions.
